# Immunological features beyond CD4/CD8 ratio values in older individuals

**DOI:** 10.18632/aging.203109

**Published:** 2021-05-26

**Authors:** Vanesa Garrido-Rodríguez, Inés Herrero-Fernández, María José Castro, Ana Castillo, Isaac Rosado-Sánchez, María Isabel Galvá, Raquel Ramos, Israel Olivas-Martínez, Ángel Bulnes-Ramos, Julio Cañizares, Manuel Leal, Yolanda María Pacheco

**Affiliations:** 1 Institute of Biomedicine of Seville (IBiS), Virgen del Rocío University Hospital (HUVR)/CSIC/University of Seville, Seville, Spain; 2 Heliopolis Nursing Home, Seville, Spain; 3 Immunovirology Unit, Internal Medicine Service, Viamed Hospital, Santa Ángela de la Cruz, Seville, Spain

**Keywords:** CD4/CD8 T-cell ratio, thymic output, inflammation, Treg, TREC

## Abstract

The CD4/CD8 T-cell ratio is emerging as a relevant marker of evolution for many pathologies and therapies. We aimed to explore immunological features beyond CD4/CD8 ratio values in older subjects (>65 years old) who were classified as having lower (<1.4), intermediate (1.4-2), or higher (>2) ratio values. The lower group showed a lower thymic output (sj/β-TREC ratio) and frequency of naïve T-cells, concomitant with increased mature T-cells. In these subjects, the CD4 T-cell subset was enriched in CD95+ but depleted of CD98+ cells. The regulatory T-cell (Treg) compartment was enriched in CTLA-4+ cells. The CD8 T-cell pool exhibited increased frequencies of CD95+ cells but decreased frequencies of integrin-β7+ cells. Interestingly, in the intermediate group, the CD4 pool showed greater differences than the CD8 pool, mostly for cellular senescence. Regarding inflammation, only hsCRP was elevated in the lower group; however, negative correlations between the CD4/CD8 ratio and β2-microglobulin and sCD163 were detected. These subjects displayed trends of more comorbidities and less independence in daily activities. Altogether, our data reveal different thymic output and immune profiles for T-cells across CD4/CD8 ratio values that can define immune capabilities, affecting health status in older individuals. Thus, the CD4/CD8 ratio may be used as an integrative marker of biological age.

## INTRODUCTION

The CD4/CD8 T-cell ratio is emerging as a relevant marker of evolution for different pathologies and therapies, including cardiovascular diseases [1], human immunodeficiency virus (HIV) infection [2, 3], and cancer [4, 5]. Furthermore, the CD4/CD8 ratio has been associated with mortality in older individuals. For example, inversion of the CD4/CD8 ratio was associated with an increased risk of mortality in Swedish OCTO and NONA longitudinal studies [6, 7]. In such cohorts, an immune risk phenotype (IRP) was mainly defined by CD4/CD8 T-cell ratio inversion but also by other T-cell alterations [6–8]. Subsequent longitudinal studies have attempted to replicate these results, with different outcomes. In a British cohort, CD4/CD8 ratio inversion was also associated with poor survival, but only before adjustment by sex [9]. Although increased mortality of individuals with an inverted T-cell ratio were confirmed in the Spanish CARRERITAS cohort [10], in the Spanish OCTABAIX study, the presence of an inverted CD4/CD8 ratio did not correlate with higher mortality in 85-year-old subjects after three years of follow-up [11]. Strikingly, in a Belgian cohort (the BELFRAIL study), a CD4/CD8 ratio above five was associated with all-cause mortality in cytomegalovirus (CMV)-seronegative very old women [12]. Despite this controversy, most studies to date reinforce the concept that alterations or imbalances in the CD4/CD8 ratio during aging may reflect clinical conditions in the elderly.

Remarkably, immune alterations related to cellular immunosenescence, together with persistent inflammation, are known to be involved in the process of deleterious aging [13, 14], which underlies the failure to maintain global health status during aging. The CD4/CD8 ratio might be related to cellular immunosenescence, and potential factors affecting the CD4/CD8 ratio in older people have been extensively studied. CMV infection has been widely reported as the main cause of CD8 T-cell oligoclonal expansion [6, 7, 15]. Additionally, free radicals, which accumulate during aging, may have an impact because subjects with an inverted CD4/CD8 ratio exhibit reduced levels of antioxidant defenses and higher oxidative stress [16]. Hence, factors associated with cumulative cellular senescence and oxidative stress appear to trigger a reduction in the CD4/CD8 ratio; however, the immunological features beyond CD4/CD8 T-cell ratio values require further exploration.

It is reasonable that CD4/CD8 T-cell ratio values, particularly in older people, might reflect different degrees of immune capabilities both for responding to novel and recall antigens and for preserving health status in this population. Although thymic output is the main regulator of T-cell homeostasis, whether it relates to the CD4/CD8 T-cell ratio in older individuals has not yet been explored. Notably, the thymus undergoes progressive atrophy throughout life, reducing its activity by approximately 3% per year until middle age, when it slows down to less than 1% per year [17, 18]. Nevertheless, the thymus remains active in adults, contributing to the renewal of the pool of naïve T-cells [19], even though thymic function is highly variable in older people [20]. In fact, intrathymic CD4^+^CD8^+^ double-positive T cells obtained from thymic biopsies correlate not only with age (negative) but also with the frequency of naïve T cells (positive) [19]. Interestingly, a relationship between thymic function and the CD4/CD8 T-cell ratio exists in HIV infection, which is a different scenario but shares several immunosenescence traits with aging [21]. On the other hand, it is also reasonable that CD4/CD8 T-cell ratio values might correlate with different inflammatory profiles. To better understand the biological meaning of the CD4/CD8 ratio in the elderly, we explored the phenotypic profiles of both CD4 and CD8 T-cells, as well as the thymic output and several inflammation-related parameters, in a population of older subjects classified according to CD4/CD8 ratio value.

## RESULTS

### Characteristics of the study population

The characteristics of the study subjects are summarized in Table 1. We analyzed 65 subjects (from 65 to 98 years old) stratified by CD4/CD8 ratio value; 35% presented a CD4/CD8 ratio lower than 1.4 and 37% a CD4/CD8 ratio higher than 2; the rest of the individuals presented intermediate values. In our cohort, ten subjects (15.4%) had an inverted CD4/CD8 ratio (<1), though only 3 of them were under 0.8, which reflects that CD4/CD8 ratio values were relatively normal in this cohort, even in the lower ratio group. Moreover, twelve subjects in this cohort had a CD4/CD8 ratio over 3 (18.5%), but only 1 showed a value over 5. Comparison groups were homogeneous regarding age, whereas a trend of fewer women in the lower CD4/CD8 ratio group than in the higher CD4/CD8 ratio group was observed (*p*=0.071). This cohort also had normal to high CD4 T-cell counts, without significant differences among the groups, but increasing levels of CD8 T-cell counts were observed in the intermediate and lower CD4/CD8 ratio groups (*p*<0.0001). No differences in other blood cell types examined were observed. All study subjects were CMV seropositive, and titers against CMV were higher in the lower CD4/CD8 ratio group than in the intermediate and higher groups (*p*=0.013 and *p*=0.065, respectively).

**Table 1:** Characterization of the study subjects.

**Variable**	**CD4/CD8<1.4** ***N* = 22** **(Group A)**	**1.4<CD4/CD8<2** ***N* = 19** **(Group B)**	**CD4/CD8>2** ***N* = 24** **(Group C)**	***P* (K-W)**	***p* (M-W)** **(A *vs*. B)**	***p* (M-W)** **(A *vs*. C)**	***p* (M-W)** **(B *vs*. C)**
**CD4/CD8 ratio**	1.05 [0.90 – 1.23]	1.75 [1.56 – 1.90]	3.03 [2.18 – 4.06]	**< 0.001**	**0.002**	**< 0.001**	**0.001**
**Age (years)**	80 [72 - 89]	76 [71 - 84]	77 [65 - 87]	0.561	0.289	0.415	0.903
**Male sex, n (%)**	10 (35.7)	11 (39.3)	7 (25)	0.071	0.536	0.361	0.058
**CD4 T-cells (cell/mm^3^)**	765 [610 - 985]	844 [730 - 1059]	898 [584 - 1285]	0.545	0.284	0.429	0.807
**CD8 T-cells (cell/mm^3^)**	689 [470 - 988]	486 [384 - 630]	270 [151 - 419]	**< 0.001**	**0.033**	**< 0.001**	**0.002**
**B lymphocytes (cell/mm^3^)**	152 [86 - 231]	104 [63 - 127]	127 [68 - 306]	0.583	0.374	1	0.412
**NK lymphocytes (cell/mm^3^)**	221 [123 - 607]	161 [66 - 230]	194 [107 - 266]	0.458	0.304	0.387	0.661
**Monocytes (%)**	6.7 [5.6 – 7.6]	6.9 [5.1 – 7.6]	6 [5.3 – 7.4]	0.606	0.948	0.391	0.398
**Neutrophils (%)**	58.4 [53.6 – 65.5]	58 [53.1 – 63.2]	62.7 [55.6 - 66]	0.398	0.565	0.416	0.187
**Eosinophils (%)**	3.6 [1.7 – 4.2]	3.4 [1.8 – 4.3]	3.2 [2.2 – 4.7]	0.983	0.824	0.955	0.919
**Basophils (%)**	0.2 [0.1 – 0.3]	0.2 [0.1 – 0.4]	0.3 [0.2 – 0.4]	0.682	0.810	0.381	0.601
**Platelets (x10^9^/L)**	244 [184 - 321]	197 [165 - 253]	212 [168 - 287]	0.214	0.089	0.239	0.533
**Anti-CMV IgG (AU/mL)**	34.4 [10.5 – 41.5]	21.8 [12.2 – 25.8]	24.3 [6.8 – 43.5]	**0.038**	**0.013**	0.065	0.549

Notes: Quantitative variables are expressed as median [IQR], and categorical variables are expressed as the number of cases (%). Variables with a *p-*value <0.05 were considered statistically significant, as shown in bold. Abbreviations: K-W, nonparametric Kruskal-Wallis test; M-W, nonparametric Mann-Whitney *U* test.

### A lower CD4/CD8 ratio is associated with lower thymic output and altered distribution of maturational T-cell subsets

**Figure 1:**
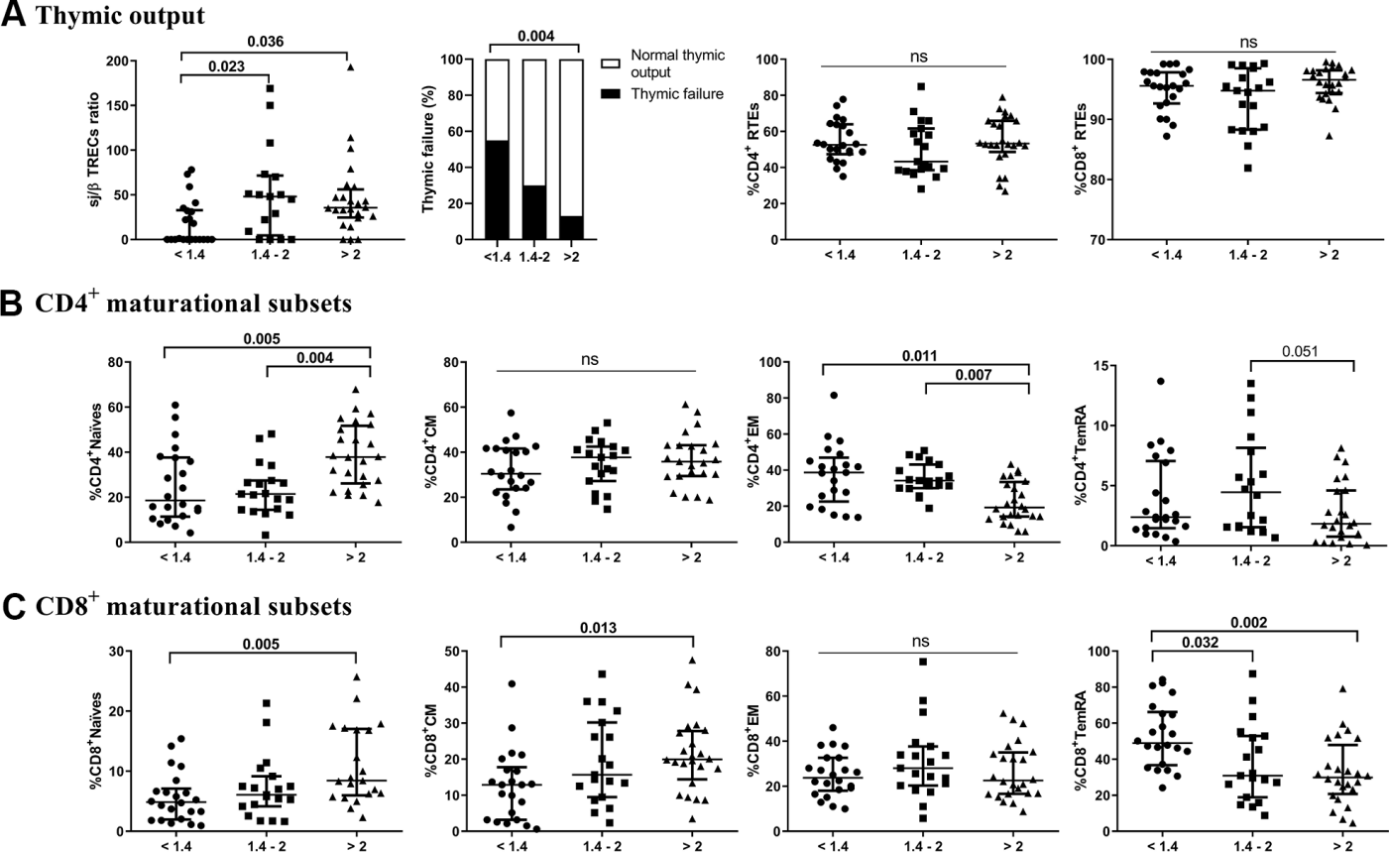
Characterization of thymic output (**A**) to the T-cell compartment and maturational subsets of CD4 (**B**) and CD8 (**C**) T-cells. The median and interquartile range (IQR) are shown. Categorical variables are expressed as the percentage of the number of cases. Subjects were classified according to a lower (1^st^ tertile, <1.4), intermediate (2^nd^ tertile, 1.4-2), or higher (3^rd^ tertile, >2) CD4/CD8 ratio. Variables with a *p-*value <0.05 were considered statistically significant, as shown in bold. TREC, T-cell receptor excision circles; RTEs, recent thymic emigrants; CM, central memory; EM, effector memory; TemRA, terminally differentiated effector memory; ns, nonsignificant.

We first quantified the thymic contribution to the T-cell compartment (Figure 1A). The lower CD4/CD8 ratio group showed a significantly lower thymic output than both the intermediate and higher ratio groups (*p*=0.023 and *p*=0.036, respectively). Additionally, we found a positive correlation between sj/β TREC and the CD4/CD8 ratio (*r*=0.305; *p*=0.015). Interestingly, 54.5% of those in the lower CD4/CD8 ratio group presented thymic failure, with only 29.4% and 12.5% in the intermediate and higher ratio groups, respectively (*p*=0.009). Nevertheless, no significant differences among the groups regarding the total number of recent thymic emigrants (RTEs) or CD4 or CD8 T cells were found. We next explored the frequencies of T-cell maturational subsets among the study groups. Regarding CD4 T-cells (Figure 1B and Supplementary Table 1), we observed a decreased frequency of naïve cells in the lower and intermediate groups (*p*=0.005 and *p*=0.004, respectively), but a more mature phenotype, namely, EM cells, appeared to accumulate in these groups (*p*=0.011 and *p*=0.007). Subjects with lower and intermediate CD4/CD8 ratios also showed a reduced CD4 naïve/memory ratio compared to those with higher CD4/CD8 ratios (Supplementary Table 1). Regarding CD8 T cells (Figure 1C and Supplementary Table 1), we observed lower frequencies of naïve and CM cells in the lower group than in the higher group (*p*=0.005 and *p*=0.013, respectively). Nevertheless, the frequency of TemRA was increased in the lower group compared to both the intermediate and higher groups (*p*=0.032 and *p*=0.002, respectively). In this subset, we did not observe any significant differences in the CD8 naïve/memory ratio between the latter groups (Supplementary Table 1). Furthermore, no differences among groups in terms of double-negative (CD4^-^CD8^-^) or double-positive (CD4^+^CD8^+^) T-cells were observed (Supplementary Table 1).

### A lower CD4/CD8 ratio is associated with altered phenotypes in the CD4 T-cell subset

**Figure 2:**
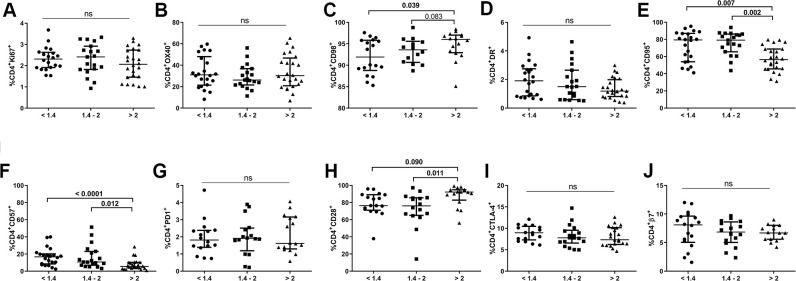
**Characterization of CD4 T-cell subsets.** Frequencies (median and IQR) of CD4 T-cells expressing proliferation (Ki67, **A**, OX40 **B**), metabolic-related (CD98, **C**), activation (HLA-DR, **D**, CD95, **E**), exhaustion/senescence (CD57, **F**, PD1, **G** CD28, **H**), suppression (CTLA-4, **I**), and gut-homing imprinting (integrin β7, **J**) markers. Subjects were classified according to a lower (1st tertile, <1.4), intermediate (2nd tertile, 1.4-2), or higher (3rd tertile, >2) CD4/CD8 ratio. Variables with a p-value <0.05 were considered statistically significant, as shown in bold. ns, nonsignificant.

We also evaluated expression of different markers of proliferation (Ki67, OX40), metabolism (CD98), activation (HLA-DR), apoptosis-prone (CD95), senescence/exhaustion (CD57, CD28 and PD1), suppressive function (CTLA-4), and gut-homing imprinting (integrin β7) in the CD4 T-cell population (Figure 2). None of the comparison groups differed in terms of the frequencies of Ki67^+^, OX40^+^, HLA-DR^+^, PD1^+^, CTLA-4^+^ or integrin β7^+^ CD4^+^ T-cells. However, we did detect a significantly lower frequency of CD98^+^CD4^+^ T-cells, an energetic metabolism-related marker, in individuals with lower CD4/CD8 ratios than in those with higher ratios (93.5[88.7-95.7] *vs.* 96.0[92.9-97.0], *p*=0.039; Figure 2C). Notably, subjects with an intermediate ratio already exhibited similar values to those in the lower ratio group (93.6[90.7-95.6]). Individuals with lower and intermediate CD4/CD8 ratios also had increased frequencies of CD4^+^CD57^+^ T-cells and decreased frequencies of CD4^+^CD28^+^ T-cells compared to subjects with a higher CD4/CD8 ratio (16.7[8.5-20.2] and 10.6[6.6-21.8] *vs*. 5.0[3.5-10.1], *p*<0.0001, *p*=0.012, respectively, for CD57; Figure 2F; 76.4[71.1-89.1] and 76.1[65.1-85.5] *vs*. 92.2[82.8-95.0], *p*=0.090, *p*=0.011; respectively, for CD28; Figure 2H). In addition, a higher frequency of CD4^+^CD95^+^ T-cells in both the lower and intermediate groups than in the higher group was found (79.4[54.0-87.0] and 79.1[65.5-86.2] *vs*. 56.4[45.7-68.8], *p*=0.007 and *p*=0.002;, respectively; Figure 2E). Interestingly, CD95 expression was increased in all CD4 maturational phenotypes in the group with a lower ratio compared to that with a higher ratio (Supplementary Figure 1A) and was statistically significant in all cellular subsets, except for EM cells (naïve, *p*=0.042; CM, *p*=0.020; EM, *p*=0.069; TemRA, *p*=0.021; respectively).

### A lower CD4/CD8 ratio is associated with higher CTLA-4-expressing Treg cells

**Figure 3:**
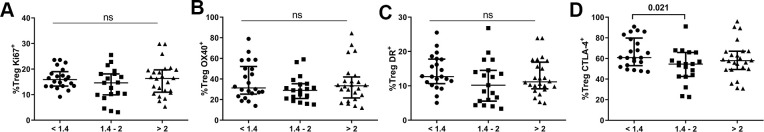
**Characterization of the Treg subset.** Frequencies (median and IQR) of Treg cells expressing Ki67 (**A**), OX40 (**B**), HLA-DR (**C**), and CTLA-4 (**D**). Subjects were classified according to a lower (1st tertile, <1.4), intermediate (2nd tertile, 1.4-2), or higher (3rd tertile, >2) CD4/CD8 ratio. Variables with a p-value <0.05 were considered statistically significant, as shown in bold. ns, nonsignificant.

Regarding the Treg population (Supplementary Table 1), there was no difference in total pool of Tregs among the groups, though the lower group presented a higher frequency of the non-Treg subset defined by Miyara’s classification [22] than the higher group (*p*=0.045). Interestingly, a nonsignificant tendency in the effector Treg pool to be higher in subjects presenting lower CD4/CD8 ratios was observed (*p*=0.072). We next examined the frequencies of total Tregs expressing several markers previously described for CD4/CD8 ratios, and neither the lower nor intermediate CD4/CD8 ratio group differed significantly from the higher ratio group in terms of Ki67, HLA-DR, or OX40 expression (Figure 3). Nonetheless, subjects with lower CD4/CD8 ratios showed an increased frequency of CTLA-4-expressing Tregs compared to the intermediate group (61.0[53.5-78.7] *vs*. 54.6[42.9-65.9], *p*=0.021).

### A lower CD4/CD8 ratio is associated with altered phenotypes in the CD8 T-cell subset

**Figure 4:**
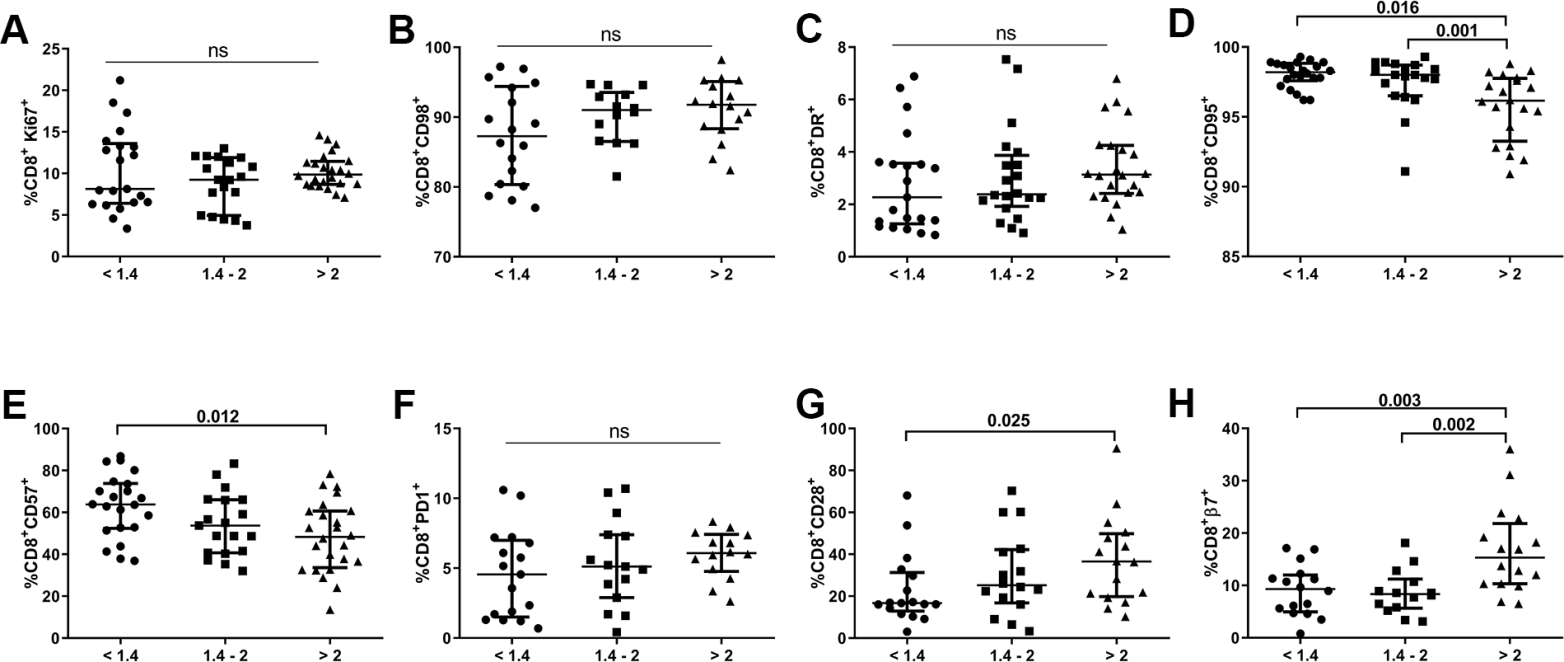
**Characterization of CD8 T-cell subsets.** Frequencies (median and IQR) of CD8 T-cells expressing proliferation (Ki67; **A**), metabolic-related (CD98; **B**), activation (HLA-DR, CD95; **C**, **D**), exhaustion/senescence (CD57, PD1, CD28; **E**–**G**), and gut-homing imprinting (integrin β7; **H**) markers according to the CD4/CD8 ratio. Subjects were classified according to a lower (1^st^ tertile, <1.4), intermediate (2^nd^ tertile, 1.4-2), or higher (3^rd^ tertile, >2) CD4/CD8 ratio. Variables with a *p-*value <0.05 were considered statistically significant, as shown in bold. ns, nonsignificant.

We also evaluated expression of the abovementioned biomarkers in the CD8 T-cell population of our cohort. As shown in Figure 4, subjects with lower CD4/CD8 ratios did not present any significant differences regarding expression of proliferation (Ki67), energetic metabolism-related (CD98), activation (HLA-DR), or apoptosis susceptibility (PD1) markers. However, we did find increased frequencies of CD8^+^CD57^+^ T-cells and decreased frequencies of CD8^+^CD28^+^ T-cells in the lower ratio group compared to those with a higher CD4/CD8 ratio (63.8[52.4-73.8] *vs*. 48.3[33.7-60.7], *p*=0.012 and 16.7[12.9-31.3] *vs.* 36.5[19.8-49.8], *p*=0.026; respectively; Figure 4E, 4G). Remarkably, we also detected a lower frequency of CD8 T-cells expressing integrin β7 in individuals with lower and intermediate CD4/CD8 ratios than in those with higher ratios (9.3[5.0-12.0] and 8.4[5.7-11.2] *vs.* 15.3 [10.3–21.8]; *p*=0.003 and *p*=0.002, respectively; Figure 4H). We also observed an increased frequency of CD8 T-cells expressing CD95 in the lower and intermediate CD4/CD8 ratio groups compared to the higher ratio group (98.2[97.6-98.8] and 98.0[96.5-98.7] *vs*. 96.2[93.3-97.8]; *p*=0.016 and *p*=0.001, respectively; Figure 4D). Similar to the CD4 subset, CD95 was highly expressed in all CD8 T-cell maturational subsets, except for EM cells, with differences between subjects having lower and higher CD4/CD8 ratios, where a trend was also observed, although nonsignificant (naïve, *p*=0.003; CM, *p*=0.009; EM, *p*=0.099; TemRA, *p*=0.008; Supplementary Figure 1B).

### A lower CD4/CD8 ratio is associated with increased levels of inflammation-related parameters and a trend toward more comorbidities

**Figure 5:**
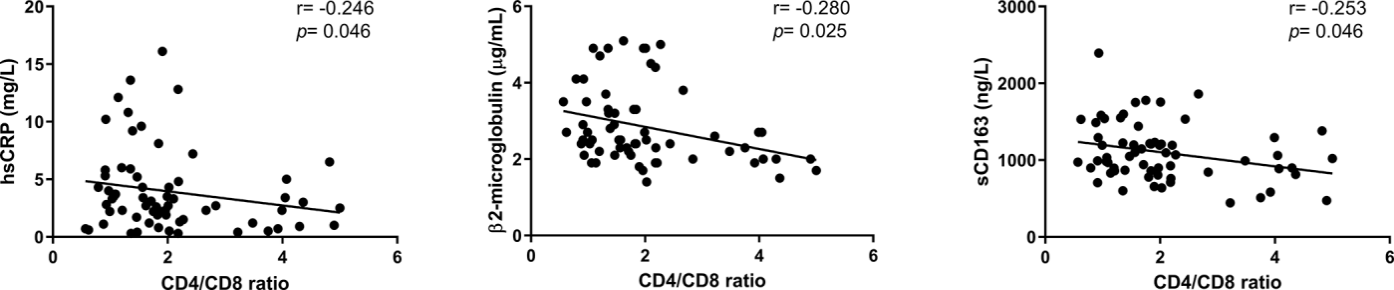
**Associations between the CD4/CD8 ratio and inflammatory-related parameters.** Correlations were assessed using Spearman’s rho correlation coefficient. Variables with a *p*<0.05 were considered statistically significant. hsCRP, high-sensitivity C reactive protein; sCD163, soluble CD163.

We finally examined potential associations between inflammation-related markers and the CD4/CD8 ratio (Supplementary Table 2). No differences regarding IL-6, a hallmark biomarker of chronic inflammatory conditions, and most of the analyzed markers were observed among the groups. However, increased levels of hsCRP were found in the lower group compared to both the intermediate and higher groups (3.70[2.23-3.33] *vs*. 2.25[1.75-3.33] and 2.30[0.90-3.4], *p*=0.040, *p*=0.046, respectively). Indeed, we observed a negative correlation between hsCRP and the CD4/CD8 ratio value (*r*=-0.246; *p*=0.046, Figure 5). Additional negative correlations between the adaptive immune activation marker, β2 microglobulin and the monocyte activation marker sCD163 and the CD4/CD8 ratio were also detected (*r*=-0.280; *p*=0.025 and *r*=-0.253; *p*=0.046, respectively; Figure 5). As inflammation and immune activation are involved in the development of many pathologies, we evaluated the presence of comorbidities in this cohort (Table 2) and found that subjects with lower and intermediate CD4/CD8 ratios exhibited a trend of more comorbidities than those presenting higher ratios (*p*=0.073 and *p*=0.083, respectively). Furthermore, subjects with a lower ratio appeared to be less independent according to the Barthel index than the intermediate and higher ratios, though only when compared to the intermediate group (*p*=0.051).

**Table 2:** Comorbidities and disability degree recorded as barthel index for functional activities of daily living for subjects classified according to CD4/CD8 ratio.

	**CD4/CD8<1.4** ***N* = 22** **(Group a)**	**1.4<CD4/CD8<2** *N* = 19* (Group b)	**CD4/CD8>2** ***N* = 24** **(Group c)**	***p* (K-W)**	***p* (M-W)** **(a *vs*. b)**	***p* (M-W)** **(a *vs*. c)**	***p* (M-W)** **(b *vs*. c)**
**Comorbidities (number)**	4 [2 - 5]	4 [2 - 5]	2 [1 -4]	0.555	0.862	0.073	0.083
**Barthel index** **Score**	70 [34 - 95]	83 [73 - 100]	83 [18 - 99]	0.183	0.051	0.464	0.343
**< 20**	3 (13.6)	1 (5.5)	4 (16.7)				
**20-35**	0 (0)	0 (0)	2 (8.3)				
**40-55**	5 (22.7)	0 (0)	0 (0)				
**≥ 60**	10 (45.5)	10 (55.5)	11 (45.8)				
**100**	4 (18.2)	7 (38.8)	7 (29.2)				

Notes: A Barthel index of 100 is assigned to a fully independent individual, whereas <20 means fully dependent. The quantitative variables, *Comorbidities* and *Score*, are expressed as the median [IQR], and categorical variables are expressed as the number of cases (%). *n=18 for Barthel index. A *p* value <0.05 was considered statistically significant. Abbreviations: K-W, nonparametric Kruskal-Wallis test; M-W, nonparametric Mann-Whitney *U* test.

## DISCUSSION

**Figure 6:**
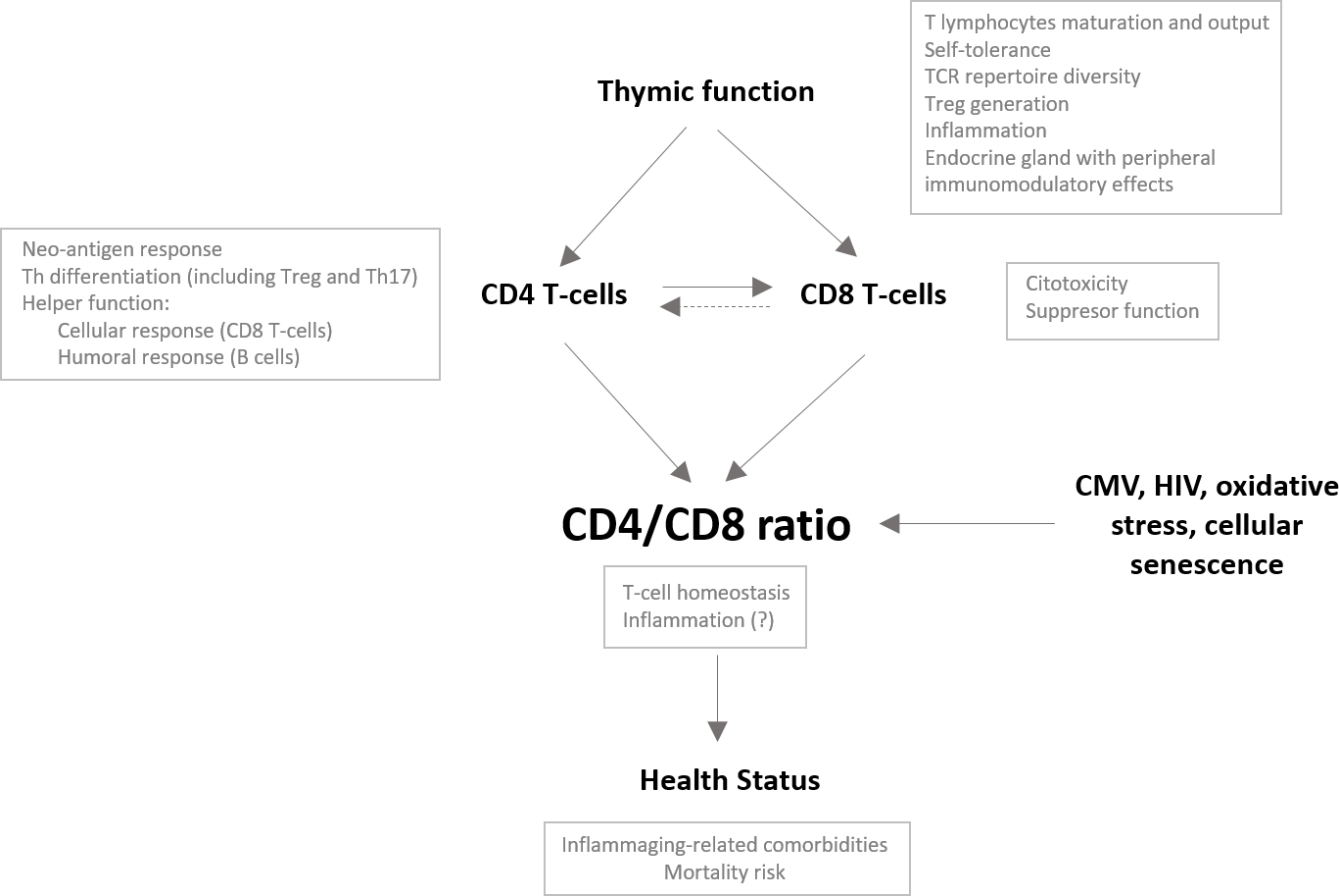
**Potential role of the thymus impacting the CD4/CD8 ratio in the older population.** The CD4/CD8 ratio may reflect the overall immune status in older people and hence be associated with the development of comorbidities and the risk of mortality in this population. Many factors have been described to impact the CD4/CD8 ratio. We propose a major role of the thymus and, specifically, its capacity to generate and maintain the functional capacities of both the CD4 and CD8 T-cell compartments; however, it is conceivable that, as an endocrine gland, the thymus also impacts the CD4/CD8 ratio through peripheral immunomodulatory effects. A proper adaptive immune response requires activation of CD4 T cells and their differentiation to helper cells, which will ultimately promote CD8 T cell activation. Adequate thymic output would guarantee the functionality of both T-cell subsets and may control clonal expansions of both subsets, probably favoring the maintenance of adequate relative proportions. Alternatively, dysfunctional helper activity of the CD4 T-cell pool due to reduced thymic function might contribute to the expansion and exhaustion of the CD8 T-cell compartment to compensate for the lack of response, leading to a reduction in the CD4/CD8 ratio.

Our study describes immunological features beyond CD4/CD8 T-cell ratio values in an older population. We show that CD4/CD8 T-cell ratio values in older people are associated with thymic output and with different phenotypes of maturational and functional CD4 and CD8 T-cell subsets. Moreover, in our study population, ratio values correlated with several parameters of innate activation and inflammation and tended to correlate with health status. Taken together, we propose a model integrating thymic function and its impact on the CD4/CD8 ratio and, hence, health status (see Figure 6).

Studies focused on emphasizing immunological status rather than chronological age have acquired great relevance in immunosenescence. Recently, Alpert et al*.* [23] described not only longitudinal alterations in immune cellular subsets throughout aging but also interindividual differences due to genetic and environmental variability that ultimately affect the immune response. Therefore, differences in CD4/CD8 ratio values might reflect some of these changes and describe the immune status more accurately than chronological age.

We first identified a potential sex-based bias among the study subjects: the higher CD4/CD8 ratio group contained by a larger population of women. This has been previously described in studies [24, 25] that attributed it to a hormonal effect and even suggested potential enhanced thymic function in women [20, 24, 26, 27]. Although such a potential link between the CD4/CD8 T-cell ratio and thymic function has not been further explored to date, the role of the thymus in the regulation of T-cell homeostasis has been well established. Similarly, Sauce et al [28] demonstrated that young adults who had undergone thymectomy during childhood and CMV-seropositive individuals exhibited an accelerated decrease in the naïve/memory T-cell ratio to levels normally found in older individuals. It is possible that induction of CMV-specific responses would exhaust the naïve T-cell pool in the absence of adequate T-cell renewal from the thymus [28]. Along this line, it is well known that expansion of CMV-specific memory CD8 T-cells [16, 29, 30] is linked to aging and that CMV impacts CD4 T-cell subsets by promoting their maturation to late differentiated cells [29]. In our population, those with lower CD4/CD8 ratios exhibited lower thymic output and more thymic failure, which was consistent with evidence regarding the naïve/memory T-cell ratio, at least for CD4, and with a higher humoral response against CMV. Interestingly, in the Leiden 85+ study, a lower proportion of naïve CD8 T-cells and a higher frequency of memory T-cells were associated with 8-year survival in subjects older than 89 years [31]. The authors argued that due to the low exposure to novel pathogens in this population, enrichment of experienced memory cells over naïve cells would provide a survival advantage to control previously encountered agents, such as CMV. Our data do not necessarily contradict those for the very old, as extensive immune remodeling is expected to occur with age, and different age strata may contextualize different immunological features.

Regarding thymic output, we also observed a decline in naïve CD4 and CD8 T-cells in subjects with lower ratios, which was even evident in the intermediate CD4/CD8 ratios for CD4, but these differences did not apparently affect RTE pools. Some studies have proposed that RTEs and naïve cells share a unique niche and that their competence for IL-7 thus determines their survival [32]. Reasonably, increasing the peripheral proliferation rate of RTEs would compensate for a lower naïve proportion. On the other hand, because RTEs contribute to maintaining the TCR repertoire [32], a longer lifespan of these cells would also amplify the RTE pool in these subjects. Alternatively, the consideration of CD31 as an RTE marker may have some intrinsic limitations due to downregulation of CD31 expression in T-cells when activated by TCR induction [33], the presence of circulating CD31^+^ T-cells in thymectomized patients [34], or the inability to discriminate between RTE and naïve T-cells generated by homeostatic proliferation [35].

Functional-related phenotypic analysis of the CD4 T-cell subset allowed us to identify a decreased frequency of CD98-expressing CD4 T-cells in subjects with lower and intermediate CD4/CD8 ratios, mainly affecting EM and CM phenotypes (data not shown). CD98 constitutes a transporter that internalizes large neutral amino acids—a source for the Krebs cycle—in exchange with internal glutamine [36]. Metabolic analysis of CD98-knockout Cal33 cells revealed that these cells had a low oxygen consumption rate, a more quiescent phenotype and, consequently, a reduced proliferation rate compared to wild-type cells [36]. This is consistent with the observed higher frequency of CD4^+^CD57^+^ cells and decreased frequency of CD4^+^CD28^+^ cells. Taken together, these results support a poorer proliferative ability of the CD4 T-cell subset in subjects with a lower CD4/CD8 ratio (and even in the intermediate ratio group), which may affect their helper function.

Regarding the Treg subset, only slight alterations in the setting of lower CD4/CD8 ratios were observed. The trend toward a higher frequency of effector Tregs in the lower CD4/CD8 ratio group, along with the increased CTLA-4 expression in the total Treg subset, suggests a more tolerant immune state in individuals with lower CD4/CD8 ratios and, hence, a poorer immune response to antigens. An increase in immune tolerance has been described throughout aging [37]; however, all groups in our study were similarly aged. Alternatively, our findings might help to explain the reported association between the CD4/CD8 ratio and vaccine responses [38–40].

Interestingly, similar to the CD4 subset, the CD8 T-cell population in the lower CD4/CD8 ratio group displayed an increased frequency of CD95-expressing cells, regardless of maturational status. Given its major role in the apoptotic process [41], membrane exposure to CD95 represents a key mechanism to remove activated and potentially harmful cells in the immune system. On the other hand, we found higher frequencies of CD8^+^CD57^+^ cells but lower frequencies of CD8^+^CD28^+^ cells in the lower CD4/CD8 ratio group. Dorneles et al [42] demonstrated not only a low frequency of CD8^+^CD28^+^ cells in aged individuals with an inverted CD4/CD8 ratio but also lower DNA damage events in peripheral lymphocytes, supporting the idea of elevated death resistance among T-cells. Furthermore, CD8^+^β7^+^ cell frequency was abnormally low in those subjects with lower and intermediate CD4/CD8 ratios, particularly for CM and EM phenotypes (data not shown). Integrin β7 is a surrogate marker of gut homing and homeostatic proliferation in CD4 T-cells [43, 44], and Rosé et al [45] have demonstrated that CD8^+^β7^+^ cells are able to efficiently eliminate a murine intestinal virus but that β7^-^ cells were ineffective. Despite not observing any changes in expression of CD98 in CD8 T-cells, we identified a reduced frequency of CD98-expressing CD8 naïve and CM T-cells in subjects with a lower CD4/CD8 ratio (data not shown). Altogether, our data suggest that CD8 T-cells present a more activated cellular status, possibly concomitant with death resistance and a limited cytotoxic function, at least with regard to intestinal pathogens, associated with lower CD4/CD8 T-cell ratios.

The CD4/CD8 ratio might reflect not only adaptive immunity but also the activation state of innate cells and inflammation, as both components of the immune system are involved in the development of most age-associated chronic diseases [46]. Regardless, specific links between the CD4/CD8 ratio and inflammation have not been described thus far. In our population, although such links were slightly evident, possibly due to the relatively normal CD4/CD8 ratios in our cohort, negative correlations were observed between the CD4/CD8 ratio and β2-microglobulin and sCD163. Furthermore, subjects with lower ratios exhibited trends of more comorbidities and less independence according to the Barthel index. These clinical trends were likely to go unnoticed due to the limited number of study subjects, and we recognize that it would be much more useful to track relevant health outcomes to better address the biological significance of our findings. Nevertheless, globally, our data also point to a potential role of thymic output in the preservation of health status in this population, and we previously reported an association between thymic function and mortality in a healthy older population, showing a direct role of the thymus in human survival [10]. Indeed, in the context of the pandemic caused by SARS-CoV-2 infection, supplementation with thymosin α1, a thymus-secreted immunomodulatory factor, has been proven to reduce the mortality of severe COVID-19 in aged patients [47].

It is remarkable that our entire older population showed a well-preserved immunological status. In fact, the intermediate and lower CD4/CD8 ratio groups presented normal CD4 levels but elevated CD8 counts, evidencing that expansion of the CD8 T-cell population is a main contributor to the reduction in the CD4/CD8 ratio. However, considering the intermediate group, the CD4 T-cell subset in comparison to the CD8 T-cell subset seemed to be more sensitive to phenotypic alterations, including cellular differentiation and replicative senescence, through a reduction in CD4/CD8 ratio values. Although we did not observe many differentially expressed markers between the groups, our results may depict immunological alterations more sensitive to slight CD4/CD8 ratio variations. Indeed, subanalysis involving subjects with CD4/CD8 <1 and CD4/CD8 >3 revealed even more pronounced differences, particularly in the maturational subsets of CD4 and CD8 T-cells (data not shown), consistent with a higher imbalance in T-cell homeostasis at these extreme points of the CD4/CD8 ratio spectrum. However, the small number of these subjects in our cohort (*n*=10 and *n*=12, respectively) prevented us from drawing a conclusion.

In summary, we report immunological features, mostly involving T-cell homeostasis, beyond CD4/CD8 ratio values in the elderly and novel evidence of the relevant role that the thymus may play in maintaining a healthy condition. Lower CD4/CD8 ratio values reflect alterations not only in CD8 T-cells but also in CD4 T-cells that seem to contribute to a permanent mildly activated state, probably by compromising an appropriate immune response and contributing to inflammation. Our findings may help in identifying novel therapeutic targets to improve the quality of life of the elderly, as favoring normal values of the CD4/CD8 T-cell ratio might improve vaccine responses and reduce the risk of infection, such as that by the novel SARS-CoV-2 virus, as well as the risk of cardiovascular disease and cancer.

## MATERIALS AND METHODS

### Study design

We conducted a retrospective study in a cohort of 65 older subjects (>65 years) from the Heliópolis Nursing Home, Seville, who did not present cognitive impairment and were able to sign the informed consent form. Blood samples were collected from October to November 2015 and processed at the Institute of Biomedicine of Seville/Virgen del Rocío University Hospital. Subjects were only included if they (a) were not on an antitumor regimen or any therapy that might affect the immune response (such as corticosteroids), (b) did not have an active infection, or (c) had not been hospitalized in the preceding 6 months. Comorbid medical conditions, including cardiovascular diseases, metabolic disorders, bone/joint diseases, brain diseases, respiratory diseases, cancer, digestive diseases, genitourinary pathologies, dermatological diseases, and habits/addictions, were recorded at the time of sample collection for all the nursing home residents included in this study (for more detail on the type of events recorded, see ref. [48]). Furthermore, we evaluated the Barthel index for functional activities of daily living (ADL) as a disability degree parameter, in which 100 is assigned to a totally independent individual and <20 indicates totally dependent. All subjects included in this study were classified into three groups according to the CD4/CD8 ratio value as follows: lower (1^st^ tertile, <1.4), intermediate (2^nd^ tertile, 1.4–2), and higher (3^rd^ tertile, >2) CD4/CD8 ratios. The study was approved by the Ethics Committee of Virgen del Rocío and Virgen Macarena University Hospitals, and all participants gave informed consent.

### Laboratory measurements and soluble biomarkers

Cell counts and percentages (lymphocytes, monocytes, neutrophils, basophils, eosinophils, and platelets) from fresh blood samples were measured with an Epics XL-MCL flow cytometer (Beckman-Coulter, Brea, California). Aliquots of serum and plasma were stored at –20° C until use. Lipopolysaccharide-binding protein (LBP; Human ELISA kit, Hycult Biotech, Uden, The Netherlands) and anti-CMV IgG antibody titers (Cytomegalovirus IgG ELISA Kit, Abnova, Taiwan, China) were measured by colorimetric enzyme-linked immunosorbent assays (ELISAs) following the manufacturer’s instructions. The following inflammation-related biomarkers were also measured. High-sensitivity C-reactive protein (hsCRP) and β2-microglobulin levels in serum were measured by an immunoturbidimetric assay using Cobas 701 (Roche Diagnostics, Mannheim, Germany). Levels of the soluble markers interleukin-6 (IL-6; Quantikine® HS ELISA, R&D Systems, Minneapolis, Minnesota) and CD163 (sCD163; MacroCD163™, IQProducts, Groningen, The Netherlands) were quantified by ELISA. The soluble biomarker D-dimer in plasma was considered an inflammatory biomarker in the absence of venous thrombosis, and levels were determined with an automated latex enhanced immunoassay (HemosIL D-Dimer HS 500, Instrumentation Laboratory, Bedford, Massachusetts). In addition, we estimated the platelet-to-lymphocyte ratio (PLR) and the neutrophil-to-lymphocyte ratio (NLR) as inflammatory indices [49].

### Flow cytometry

Peripheral blood mononuclear cells (PBMCs) were isolated from fresh blood samples and cryopreserved until analysis. After thawing, one million PBMCs from each sample was immunophenotyped by staining with the following surface antibodies: anti-CD31 PE-CF594, anti-CD3 APC-H7, anti-CD4 BV786, anti-CD8 PerCP-Cy5.5, anti-CD25 BV605, anti-CD45RA BV650, anti-PD-1 BV650, anti-CD98 BV421 (BD Biosciences, USA); anti-CD57 PE-Cy7, anti-HLA-DR BV570, anti-CD95 BV711, anti-CD27 AF700 (BioLegend, USA); anti-Beta7 FITC (eBioscience, Thermo Fisher Scientific, USA) and anti-CD28 PE (Beckman Coulter, USA). Viable cells were identified using LIVE/DEAD fixable Aqua Blue Dead Cell Stain (Life Technologies, USA). For intracellular staining, the cells were then fixed and permeabilized according to the manufacturer’s instructions (FoxP3/Transcription Factor Staining Buffer, eBioscience, USA) and stained with anti-Ki67 PerCP-Cy5.5, anti-FoxP3 PE, and anti-CTLA4 APC antibodies (BD Biosciences, USA). Flow cytometry was performed using an LSR Fortessa (BD, USA), and a minimum of 100,000 total lymphocyte events were acquired from each sample. The analysis was performed with FlowJo version 9.2 (TreeStar), and the data are expressed as frequencies (%).

We determined CD4 and CD8 T-cell maturation subsets, as defined as naïve (CD27^+^CD45RA^+^), central memory (CM; CD27^+^CD45RA^-^), effector memory (EM; CD27^-^CD45RA^-^), terminally differentiated effector memory (TemRA; CD27^-^CD45RA^+^), and recent thymic emigrants (RTEs; naïve CD31^+^). We also identified the total Treg cell pool (CD25^hi^FoxP3^+^) and classified cells into different functional subsets as reported by Miyara et al [22]: naïve Tregs (CD45RA^+^FoxP3^lo^), effector Tregs (CD45RA^-^FoxP3^hi^), and non-Tregs (CD45RA^-^FoxP3^lo^). Characterization of the maturational subsets was performed by measuring expression of the following functional-related markers: proliferation (Ki67, OX40), energetic metabolism-related (CD98), activation (HLA-DR), apoptotic susceptibility (CD95), senescence/exhaustion (CD28, CD57, PD-1), suppression (CTLA-4), and gut-homing imprinting (integrin β7). Our gating strategy has already been reported in Herrero-Fernández et al*.* [48].

### sj/β TREC ratio quantification

Thymic output was analyzed by using DNA from PBMCs with qPCR quantification of the sj/β T-cell receptor excision circles (TREC) ratio. This technique has been previously optimized and published elsewhere [50]. Briefly, sj-TREC were amplified in a PCR reaction tube; the six DβJβ-TREC from cluster one were amplified together in a different PCR reaction tube. To guarantee correct quantification at the real-time PCR step, twenty-one amplification rounds were performed. All amplicons (DβJβ- and sj-TREC) were then amplified together in a second round of PCR using a LightCycler® 480 System (Roche Molecular Biochemicals, Mannheim, Germany). Thymic failure is defined as sj/β TREC ratio lower than 10; it has been associated with the survival index in a cohort of older adults [10].

### Data analysis

Quantitative variables are expressed as median and interquartile range [IQR]. Statistical analysis between groups was performed using a nonparametric Kruskal-Wallis H test; when considered significant (p<0.05), multiple comparisons between different groups were carried out using nonparametric Mann-Whitney U tests. Categorical variables were recorded as the number of cases and percentages, with comparisons among groups using the χ2 or Fischer’s exact test. Correlations were assessed using Spearman’s rho correlation coefficient. A *p*-value<0.05 was considered statistically significant. The statistical analysis was performed using Statistical Package for the Social Sciences software (SPSS version 25; IBM SPSS, Chicago, USA). Graphs were created using Prism (version 8, GraphPad Software, Inc., USA).

## Supplementary Material

Supplementary Figure 1

Supplementary Tables
