# Supplementary material for: Immunological features beyond CD4/CD8 ratio values in older individuals
Source: Aging (Albany NY). 2021 May 26;13(10):13443–59. doi: 10.18632/aging.203109 (PMC8202849; doi:10.18632/aging.203109)
Supplement: Supplementary Figure 1 [file aging-13-203109-s001.pdf]

SUPPLEMENTARY FIGURE

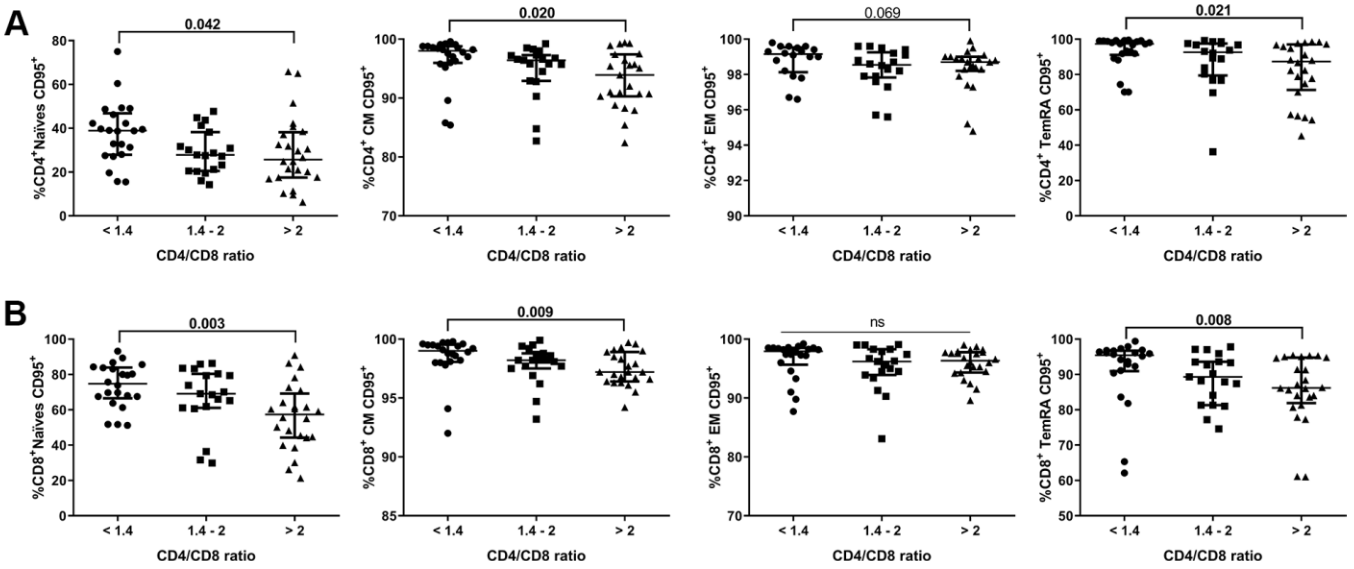

Supplementary Figure 1. Maturational subsets of CD4 (**A**) and CD8 (**B**) T-cells expressing the apoptosis-prone marker CD95 according to CD4/CD8 ratio. Comparisons between medians (IQR) with a p value <0.05 were considered statistically significant and are shown in bold. CM, central memory; EM, effector memory; TemRA, terminally differentiated effector memory.
