# Supplementary material for: Immunological features beyond CD4/CD8 ratio values in older individuals
Source: Aging (Albany NY). 2021 May 26;13(10):13443–59. doi: 10.18632/aging.203109 (PMC8202849; doi:10.18632/aging.203109)
Supplement: Supplementary Tables [file aging-13-203109-s002.pdf]

SUPPLEMENTARY TABLES

Supplementary Table 1. Maturational T-cell subsets stratified by lower (1st tertile, <1,4), intermediate (2nd tertile, 1,4-2) or higher (3rd tertile, >2) CD4/CD8 ratios.

Cell subset	CD4/CD8<1.4 *N* = 22 (Group a)	1.4<CD4/CD8<2 *N* = 19 (Group b)	CD4/CD8>2 *N* = 24 (Group c)	*p* (K-W)	*p* (M-W) (a *vs.* b)	*p* (M-W) (a *vs.* c)	*p* (M-W) (b *vs.* c)
**CD4 (%)**	47.1 [42.9 – 56.2]	60.2 [55.1 – 63.8]	73.0 [69.1 – 80.3]	**< 0.001**	0.151	**< 0.001**	**0.001**
**Naïve**	18.6 [11.4 – 37.7]	21.4 [14.4 – 27.4]	37.9 [26.1 – 51.7]	**0.001**	1	**0.005**	**0.004**
**CM**	30.5 [23.5 – 41.6]	37.7 [27.2 – 42.5]	35.5 [29.3 – 43.1]	0.548	0.425	0.291	0.922
**EM**	38.4 [19.6 - 45]	34.2 [30 – 43.1]	19.3 [14.3 - 334]	**0.002**	1	**0.011**	**0.007**
**TemRA**	2.4 [1.5 – 7.1]	4.7 [1.6 – 9.1]	1.8 [0.8 – 4.6]	0.114	0.411	0.169	**0.050**
**Naïve/CM ratio**	0.53 [0.39 – 0.99]	0.67 [0.43 – 0.84]	0.92 [0.71 – 1.87]	**0.003**	0.859	0.013	**0.010**
**Treg ($CD25^{hi}$ $FoxP3^{+}$, %)**	1.2 [1 – 1.9]	1.43 [1.2 – 1.9]	1.1 [0.8 – 1.5]	0.108	0.410	0.150	**0.049**
**Treg/Tconv ratio**	0.0122 [0.0099 – 0.0194]	0.0145 [0.0120 – 0.0195]	0.0109 [0.0077 – 0.0151]	0.108	0.410	0.150	**0.050**
**Treg (Miyara's, %):**							
**Effector**	3.3[2.3 – 5.2]	2.9 [2.5 – 4.3]	2.3 [1.4 – 4.15]	0.169	0.768	0.072	0.176
**Naïve**	5.3 [4.1 - 8]	6.4 [3.1 – 9.7]	7.2 [5.1 – 8.7]	0.368	0.638	0.146	0.456
**Non-Treg**	25.5 [16.5 – 31.6]	20.3 [18.8 – 28.6]	20.3 [11.2 – 23.3]	0.119	0.619	**0.045**	0.171
**CD8 (%)**	52.4 [43.5 – 56.6]	39.4 [34.0 – 77.5]	26.8 [19.2 – 29.7]	**< 0.001**	0.154	**< 0.001**	**0.001**
**Naïve**	4.9 [2 – 7.1]	6.1 [4.2 – 9.2]	8.4 [6 – 17.1]	**0.002**	0.987	**0.002**	0.067
**CM**	12.9 [3.2 – 17.8]	15.7 [9.5 – 30.2]	20 [14.4 – 27.8]	**0.014**	0.168	**0.013**	1
**EM**	23.8 [18 – 32.6]	28 [20.3 – 37.7]	22.6 [16.7 – 35.1]	0.478	0.261	0.869	0.322
**TemRA**	25.5 [16.5 – 31.6]	20.3 [18.8 – 28.6]	20.3 [11.2 – 23.3]	**0.002**	**0.032**	**0.002**	1
**Naïve/CM ratio**	0.68 [0.45 – 0.99]	0.59 [0.28 – 1.18]	0.92 [0.51 – 1.37]	0.198	0.123	0.140	0.753
**$CD4^{+}$ $CD8^{+}$ (%)**	0.46 [0.28 – 0.68]	0.28 [0.18 – 0.54]	0.45 [0.14 – 0.77]	0.533	0.175	0.817	0.680
**$CD4^{-}$ $CD8^{-}$ (%)**	4.95 [3.63 – 7.47]	4.63 [2.24 – 7.50]	5.82 [3.52 – 11.70]	0.656	0.586	0.390	0.300

Notes: Quantitative variables are expressed as median [IQR]. Variables with a p value <0.05 were considered statistically significant and are shown in bold. Abbreviations: K-W, non-parametric Kruskal-Wallis test; M-W, non-parametric Mann-Whitney U test; CM, central memory; EM, effector memory; TemRA, terminally differentiated effector memory; Treg, regulatory T-cell; and Tconv, conventional T-cells.

Supplementary Table 2. Parameters related to inflammatory response stratified by a lower (1st tertile, <1,4), intermediate (2nd tertile, 1,4-2) or higher (3rd tertile, >2) CD4/CD8 ratio.

Variable	CD4/CD8<1.4<br>N = 22<br>(Group a)	1.4<CD4/CD8<2<br>N = 19<br>(Group b)	CD4/CD8>2<br>N = 24<br>(Group c)	p (K-W)	p (M-W)<br>(a vs. b)	p (M-W)<br>(a vs. c)	p (M-W)<br>(b vs. c)
**IL-6 (pg/mL)**	3.71 [2.65 – 5.78]	3.10 [2.29 – 4.46]	3.36 [2.35 – 4.53]	0.402	0.124	0.474	0.762
**hsCRP (mg/L)**	3.70 [2.23 – 3.33]	2.25 [1.75 – 3.33]	2.30 [0.90 – 3.4]	0.101	**0.040**	**0.046**	0.805
**PLR**	117 [80 – 175]	93 [77 – 159]	117 [100 – 162]	0.409	0.315	0.949	0.198
**NLR**	2.22 [1.74 – 2.77]	1.92 [1.64 – 2.70]	2.47 [1.89 – 2.90]	0.421	0.693	0.330	0.246
**LBP (ng/mL)**	13.2 [10.6 – 14.9]	12.4 [10.9 – 14.5]	12.7 [9.3 – 14.7]	0.662	0.560	0.386	0.838
**β2M (μg/mL)**	2.85 [2.40 – 3.80]	2.45 [2.17 – 3.23]	2.30 [1.93 – 3.53]	0.121	0.141	0.066	0.401
**D-dimers (μg/L)**	830 [505 - 1225]	780 [410 - 1160]	660 [420 - 1220]	0.878	0.723	0.646	0.866
**sCD163 (ng/L)**	1012 [889 – 1534]	1147 [880 – 1223]	958 [724 – 1204]	0.253	0.960	0.237	0.169

Notes: Quantitative variables are expressed as median [IQR]. Variables with a *p* value <0.05 were considered statistically significant and are shown in bold. Abbreviations: K-W, non-parametric Kruskal-Wallis test; M-W, non-parametric Mann-Whitney *U* test; IL-6, interleukin 6; hsCRP, high sensitive C reactive protein; PLR, platelets to lymphocytes ratio; NLR, neutrophils to lymphocytes ratio; LBP, lipopolysaccharide binding protein; β2M, β2 microglobulin.
